# Supplementary material for: Changes in Ammonia-Oxidizing Archaea and Bacterial Communities and Soil Nitrogen Dynamics in Response to Long-Term Nitrogen Fertilization
Source: Int J Environ Res Public Health. 2022 Feb 26;19(5):2732. doi: 10.3390/ijerph19052732 (PMC8910298; doi:10.3390/ijerph19052732)
Supplement: Supplementary file 1 [file ijerph-19-02732-s001.zip › ijerph-1572418-supplementary.pdf]

International Journal of
Environmental Research and Public Health

MDPI

Supplementary material

Changes in ammonia-oxidizing archaea and bacteria community and nitrogen uptake of wheat in response to long term nitrogen fertilization

Aixia Xu [1,2], Lingling Li [1,2*], Junhong Xie [1,2], Subramaniam Gopalakrishnan [3], Renzhi Zhang [1,4], Zhuzhu Luo [1,4], Liqun Cai [1,4], Chang Liu [1], Linlin Wang [1,2], Sumera Anwar [5], Yuji Jiang [6,*]

[1] Gansu Provincial Key Laboratory of Aridland Crop Science, Gansu Agricultural University, Lanzhou 730070, China; xuax@gsau.edu.cn (A.X.); xiejh@gsau.edu.cn (J.X.); zhangrz@gsau.edu.cn (R.Z.); luozz@gsau.edu.cn (Z.L.); cailq@gsau.edu.cn (L.C.); liuc@gsau.edu.cn (C.L.); wangll@gsau.edu.cn (L.W.)
2 College of Agronomy, Gansu Agricultural University, Lanzhou 730070, China
[3] International Crops Research Institute for the Semi-Arid Tropics (ICRISAT), Patancheru, Hyderabad, Telangana 502324, India; s.gopalakrishnan@cgiar.org (S.G.)
[4] College of Resource and Environment, Gansu Agricultural University, Lanzhou 730070, China
[5] Institute of Molecular Biology and Biotechnology, The University of Lahore, Pakistan; anwer_sumera@yahoo.com (S.A.)
[6] State Key Laboratory of Soil and Sustainable Agriculture, Institute of Soil Science, Chinese Academy of Sciences, Nanjing 210008, China

* Correspondence: lill@gsau.edu.cn; yjjiang@issas.ac.cn（Y.J.）

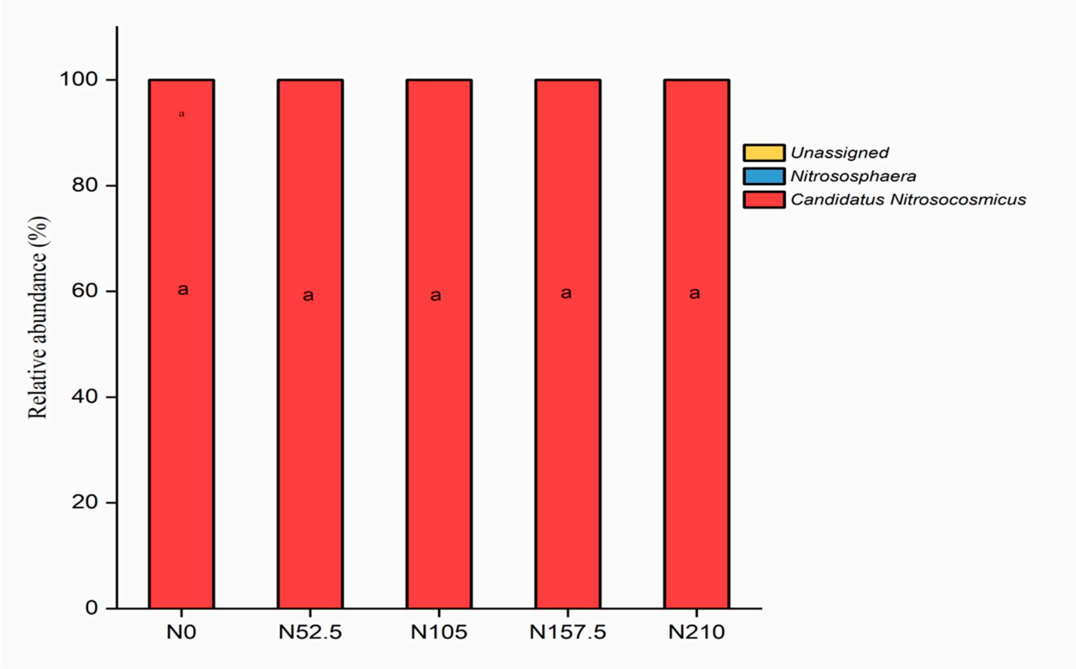

(a)

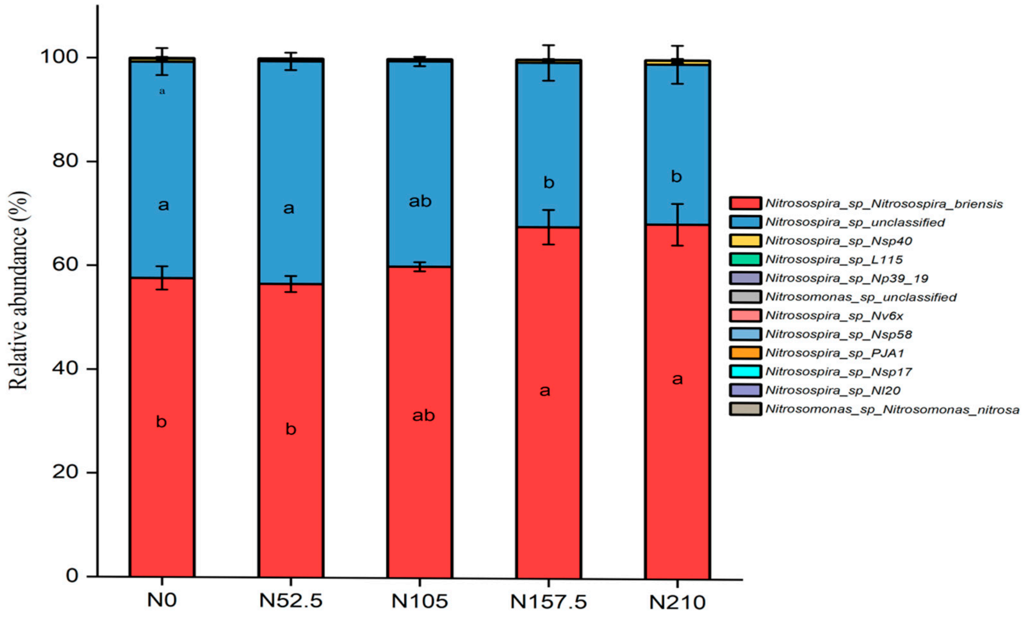

(b)

Figure S1. Relative abundance of AOA genera (a) and AOB genera (b) in the soils as affected by long-term nitrogen (N) fertilizer treatment. N0, non-N-fertilized control; N52.5, N105, N157.5, N210, annual N fertilizer application at 52.5, 105.0, 157.5, and 210.0 kg N ha-1, respectively. Error bars indicate the standard errors of the means (n = 3). Different letters indicate means that are significantly different at $P < 0.05$.

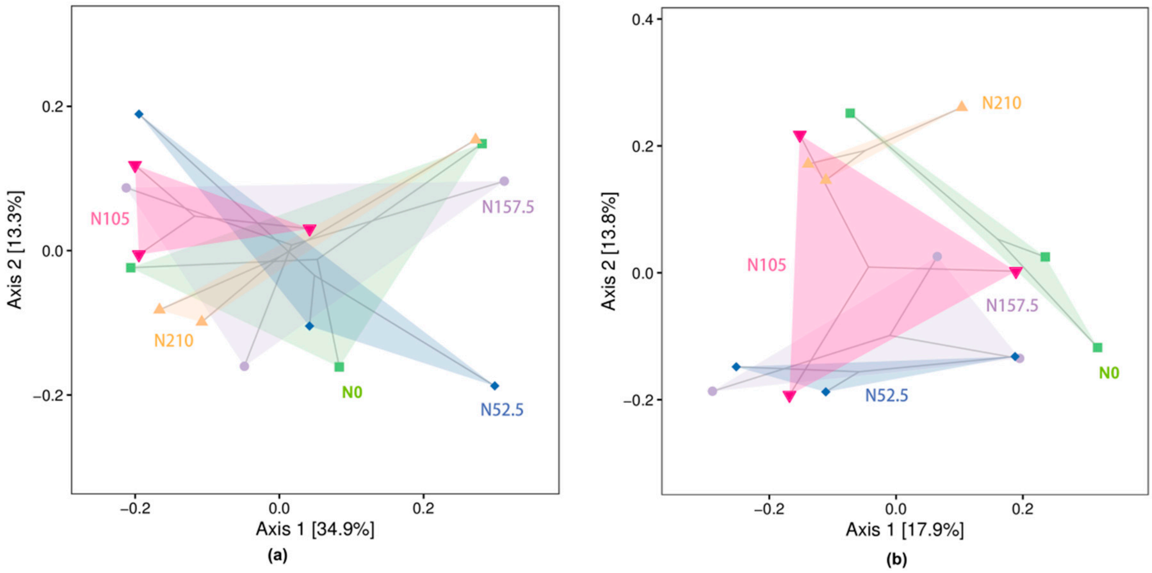

Figure S2. Principal coordinate analysis (PCoA) of AOA (a) and AOB (b) as affected by long-term nitrogen (N) fertilizer treatment. N0, non-N-fertilized control; N52.5, N105, N157.5, N210, annual N fertilizer application at 52.5, 105.0, 157.5, and 210.0 kg N ha-1, respectively.
